# Tailoring High Energy Storage Density by a Temperature-Induced Relaxor-to-Ferroelectric Phase Transition

**DOI:** 10.3390/nano16130802

**Published:** 2026-06-29

**Authors:** Qiang Lv, Jieyu Chen

**Affiliations:** 1 College of Science, Inner Mongolia University of Technology, Hohhot 010051, China; 202320908044@imut.edu.cn; 2 Discharge Plasma and Functional Materials Application Laboratory, Inner Mongolia University of Technology, Hohhot 010051, China; 3 Inner Mongolia Key Laboratory of New Materials and Surface Engineering, School of Materials Science and Engineering, Inner Mongolia University of Technology, Hohhot 010051, China

**Keywords:** dielectric energy storage, crystallization temperature, films, phase transition

## Abstract

Crystallization temperature was tuned to control the crystal structure and relaxor behavior of Na_0.5_Bi_5.5_Ti_4_AlO_18_ films. This established a structure–property regulation pathway, enabling controlled transitions from ferroelectric, non-ergodic relaxor to ergodic relaxor states. Precise crystallization temperature control reduced grain size, thereby increasing both bulk resistivity and breakdown strength via suppressed conduction pathways. The Na_0.5_Bi_5.5_Ti_4_AlO_18_ film achieves outstanding energy storage performance when crystallized at 500 °C, delivering a recoverable energy density of 49.6 J/cm^3^ and an energy efficiency of 73.5% at an applied electric field of 2820 kV/cm. It also exhibits excellent thermal and frequency stability. Thus, crystallization temperature control is a direct, effective lever for optimizing dielectric energy storage films.

## 1. Introduction

Dielectric energy storage films (DESFs) have attracted considerable attention from both academic and industrial communities owing to their high power density and exceptional thermal and electrical stability—properties that render them highly suitable for pulsed power applications [1,2]. Enhancing the energy storage performance of DESFs requires simultaneous optimization of energy storage density (*U*_re_) and efficiency (η), governed by the fundamental relationships expressed in equations $U_{\mathrm{re}}=\int_{P_{r}}^{P_{\max}}\mathrm{EdP}$ and $\eta =U_{\mathrm{re}}/U_{\mathrm{re}}+U_{\mathrm{loss}}$ [3,4,5,6,7]. Herein, under ideal conditions, simultaneously optimizing the DESFs’ maximum polarization (*P*_max_), residual polarization (*P*_r_), and breakdown strength (*E*_b_) represents the most effective strategy for achieving high energy storage density. However, a critical bottleneck in the practical deployment of DESFs is their *U*_re_, which arises from the intrinsic trade-off between *P*_max_ and *E*_b_. Liu et al. realized a high energy storage density under moderate-to-low electric fields by engineering defect dipoles and tailoring their polarization behavior [8]. Sun et al. boosted the BNBT film’s energy storage density to 86 J/cm^3^ at 1700 kV/cm by engineering super-tetragonal nanostructures near the morphotropic phase boundary [9]. In contrast, enhanced energy storage density under high electric fields is commonly associated with elevated breakdown strength and delayed saturation polarization [2,10]. Tang et al. achieved high energy storage performance via La^3+^ doping, which widens the bandgap and suppresses domain interactions, enhancing both breakdown strength and relaxor ferroelectric(RFE) behavior [11]. Yang et al. achieved outstanding energy storage performance via multi-element doping, enabled entropy regulation in thin films [10]. Chemical heterogeneity provides a versatile strategy for concurrently tuning *P*_max_, *E*_b_, and RFE behavior, thereby synergistically enhancing energy storage performance [10,11]. Energy storage performance can be regulated through multiple pathways beyond lattice modulation induced by chemical heterogeneity. Crystallization temperature serves as an alternative lattice modulation parameter that concurrently governs polarization magnitude, breakdown strength, and relaxor ferroelectric behavior, thereby enabling precise control over energy storage performance [12,13]. Cheng et al. tuned the monoclinic and tetragonal phase fractions and optimized nanoscale polar region size in KNN thin films by engineering thermal treatment parameters [12]. Hu et al. attained high energy storage density in PbZrO_3_ thin films by flash annealing, driven control of domain structure, and grain boundaries [13]. Therefore, crystallization temperature, being a critical processing parameter governing energy storage performance, warrants systematic investigation and deliberate optimization.

Na_0.5_Bi_5.5_Ti_4_AlO_18_ (NBTAO) relaxor ferroelectric films were rationally engineered by incorporating BiAlO_3_ perovskite structural motifs into the perovskite sublayers of Na_0.5_Bi_4.5_Ti_4_O_15_ ferroelectric films. As a representative bismuth-layer-structured ferroelectric (BLSF) film, it consists of periodically alternating perovskite layers and insulating layers [14,15]. Its general chemical formula is (Bi_2_O_2_)^2+^(A*_m_*_−1_B*_m_*O_3*m*+1_)^2−^, where *m* denotes the number of consecutive perovskite octahedral layers within the crystallographic unit cell. Crystallization temperature tuning enables a reversible from the ferroelectric state to the non-ergodic relaxor state to the ergodic relaxor state transition in NBTAO films, as confirmed by temperature-dependent dielectric properties and polarization-electric field (*P*–*E*) hysteresis measurements. Moreover, crystallization temperature serves as a critical parameter for tuning both grain size and carrier type, thereby enabling precise control over the material’s insulating behavior. Enhancing the *E*_b_ facilitates concurrent modulation of polarization magnitude, thereby postponing the onset of polarization saturation. This work establishes crystallization temperature-mediated synergistic control over the NBTAO film’s *E*_b_, RFE behavior, and *P*_max_. The concurrent enhancement of *E*_b_ and RFE behavior, coupled with the postponement of polarization saturation, synergistically improves the energy storage performance of the NBTAO film. The NBTAO thin film exhibits a maximum energy storage density of 49.6 J/cm^3^ and an energy storage efficiency of 73% at a crystallization temperature of 500 °C. This corresponds to a twofold increase in energy storage density relative to the sample crystallized at 650 °C. The energy storage performance remains stable over a broad temperature range (20–160 °C) and across a wide frequency spectrum (1–100 kHz), demonstrating exceptional stability and dynamic robustness. Crystallization temperature serves as an effective and experimentally accessible parameter for tuning energy storage performance, thereby offering mechanistic insights and design principles for high-performance energy storage devices.

## 2. Experimental Section

The NBTAO films were fabricated via a sol-gel process integrated with oxygen plasma treatment and subsequently annealed at 450 °C, 500 °C, 550 °C, 600 °C, and 650 °C. NBTAO-450, NBTAO-500, NBTAO-550, NBTAO-600, and NBTAO-650 denote the NBTAO films annealed at 450 °C, 500 °C, 550 °C, 600 °C, and 650 °C, respectively. The plasma treatment optimizes film growth, minimizes defects, and increases uniformity [3,4,5,6].

**Figure 1:**
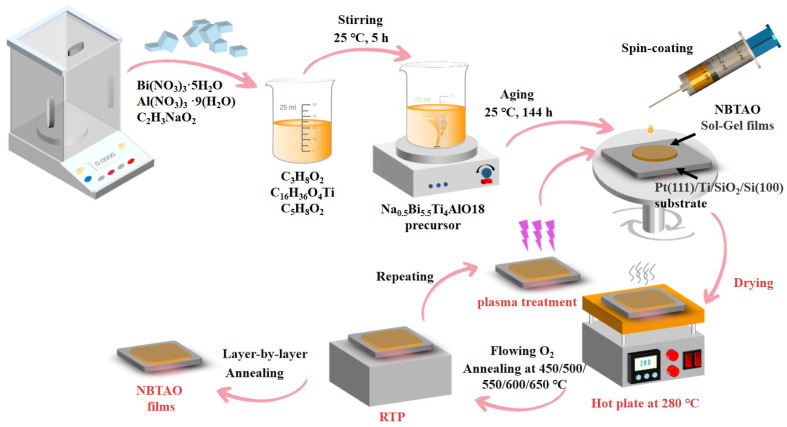
The flow chart of NBTAO precursor solution and layer-by-layer annealing with oxygen ions plasma treatment.

Further experimental details are provided in Figure 1, while the chemical reagents and instrumentation used are listed in Table 1 and Table 2, respectively. A 0.05 mol/L Na_0.5_Bi_5.5_Ti_4_AlO_18_ precursor solution is spin-coated onto Pt(111)/Ti/SiO_2_/Si(100) substrate at a rate of 7400 r/min. The wet film was dried (280 °C, 180 s, air) on a hot plate and then rapidly annealed at different temperatures (450 °C, 500 °C, 550 °C, 600 °C, and 650°, 180 s, O_2_) to crystallize. The Na_0.5_Bi_5.5_Ti_4_AlO_18_ film was fabricated by a layer-by-layer annealing technique. Each layer of Na_0.5_Bi_5.5_Ti_4_AlO_18_ film was treated with oxygen ion plasma for 180 s. Finally, the layer-by-layer annealing technique was repeated, and each layer was treated with oxygen ion plasma until the desired thickness of the Na_0.5_Bi_5.5_Ti_4_AlO_18_ film was reached.

## 3. Results and Discussion

The structure information of the NBTAO films were analyzed employing X-ray diffraction and Raman spectroscopy. The analysis of the cross section was conducted using scanning electron microscopy (SEM). Disc-shaped electrodes with an area of 5 × 10^−5^ cm^2^ were fabricated on the surface using magnetron sputtering equipment with a custom mask. A high-temperature dielectric testing system was employed to measure the temperature and electric field dependence of the dielectric constant and dielectric loss of the film. Polarization-electric field (*P*–*E*), Pund, polarization–current (*P*–*C*) curves and leakage current were evaluated with a multiferroic testing system. The LCR digital bridge coupled with a high-temperature probing stage characterizes the impedance properties.

**Figure 2:**
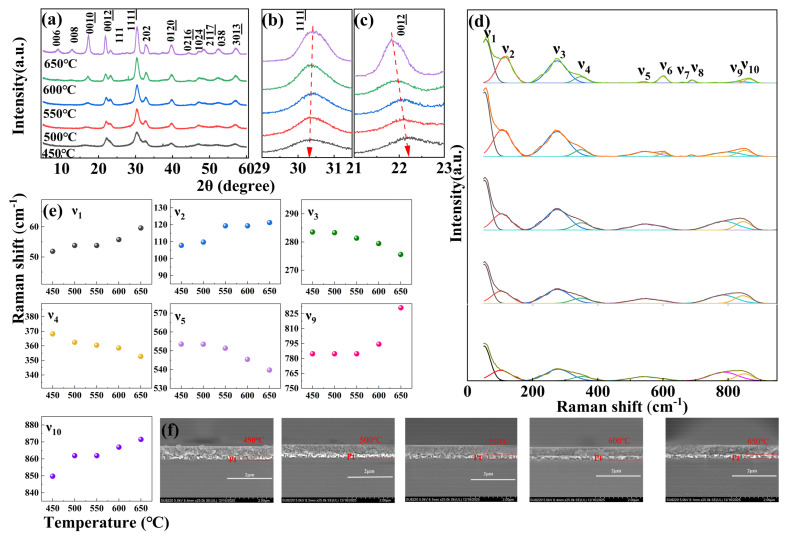
(**a**) XRD pattern of NBTAO films. (**b**,**c**) Enlarged (1111) and (0012) diffraction peaks. (**d**) Raman spectra of NBTAO films. (**e**) Raman shifts of NBTAO films as a function of temperature. (**f**) SEM cross-sectional image of the NBTAO film.

Figure 2 displays the XRD patterns of the NBTAO-450, NBTAO-500, NBTAO-550, NBTAO-600, and NBTAO-650 films. XRD confirms a pure five-layer BLSF structure, matching ICSD-150401 [4]. With decreasing crystallization temperature, the (1111) diffraction peak shifts to lower angles, whereas the (0012) diffraction peak shifts to higher angles, consistent with anisotropic lattice distortion characterized by *a*- or *b*-axis expansion and *c*-axis contraction. The full width at half maximum (FWHM) of the diffraction peaks increases monotonically as crystallization temperature decreases, consistent with grain refinement. The SEM surface images and grain-size distributions of NBTAO films at the corresponding annealing temperatures are presented in Figure S1. As can be observed from the figure, the grain size gradually diminishes and becomes finer as the annealing temperature decreases. Decreasing crystallization temperature induces diffraction peak merging and disappearance, indicating enhanced structural symmetry and a low- to high-symmetry transformation. The coexistence of crystalline-amorphous coexistence is observed in the NBTAO-450 film. Figure 2d displays the Raman shifts of the NBTAO-450, NBTAO-500, NBTAO-550, NBTAO-600, and NBTAO-650 films. The NBTAO-450, NBTAO-500, NBTAO-550, NBTAO-600, and NBTAO-650 films exhibit seven distinct vibrational modes. The *ν*_1_ and *ν*_2_ Raman modes originate from the vibrational motions of Bi^3+^ ions [3,14,15]. The Raman modes at *ν*_3_ and *ν*_4_ are assigned to torsional bending vibrations of the BO_6_ octahedra, whereas those at *ν*_9_ and *ν*_10_ arise from stretching vibrations along the BO_6_ octahedral chains linking adjacent (Bi_2_O_2_)^2+^ layers [16].

Although Raman spectroscopy offers indirect evidence of structural evolution in the form of a superposition of multiple vibrational modes, the observed peak shifts and broadening serve as macroscopic manifestations of microscopic lattice dynamics, including BO_6_ octahedral distortions and cation displacements. These spectral alterations are intricately associated with lattice strain and a reduction in long-range order. With decreasing crystallization temperature, the *ν*_1_ and *ν*_2_ Raman modes exhibit a red shift, indicating a reduction in their vibrational frequencies, arising from *a*- or *b*-axis expansion. Moreover, softening of the *ν*_2_ Raman mode is evident in Figure 2e, indicating a progressive reduction in the orthorhombicity of the NBTAO films [17]. With decreasing crystallization temperature, the Raman-active modes *ν*_3_ and *ν*_4_ exhibit a systematic blue shift, i.e., a shift to higher wavenumbers, reflecting an increase in the vibrational frequency of the O-Ti-O bending mode within the BO_6_ octahedra. This trend implies a reduction in the magnitude of octahedral tilting distortions and a concurrent relaxation of the overall orthorhombic lattice distortion [18,19]. Moreover, a systematic blue shift of the *ν*_5_ Raman peak is observed upon decreasing the crystallization temperature, signifying an increase in the vibrational frequency of the three-dimensional torsional-bending coupled mode within the BO_6_ octahedra. This frequency upshift implies an enhancement of the effective force constant associated with this mode, thereby indicating a pronounced attenuation of torsional distortion in the BO_6_ octahedral [20,21,22]. The pronounced redshifts observed in the *ν*_9_ and *ν*_10_ vibrational modes are likely attributable to contraction along the *c*-axis [23]. With decreasing crystallization temperature, the Raman spectra of the NBTAO films exhibit systematic broadening-indicative of reduced lattice long-range order, diminished grain size, and enhanced RFE behavior [24,25]. Meanwhile, as crystallization temperature decreases, certain Raman modes merge and vanish, reflecting reduced crystallinity and degraded long-range lattice order in the NBTAO films. Figure 2f shows the SEM cross-sectional image of NBTAO films, demonstrating a fully dense microstructure without observable pores or interfacial voids. The thicknesses of NBTAO-450, NBTAO-500, NBTAO-550, NBTAO-600, and NBTAO-650 films are 432, 390, 406, 395, and 402 nm, respectively.

**Figure 3:**
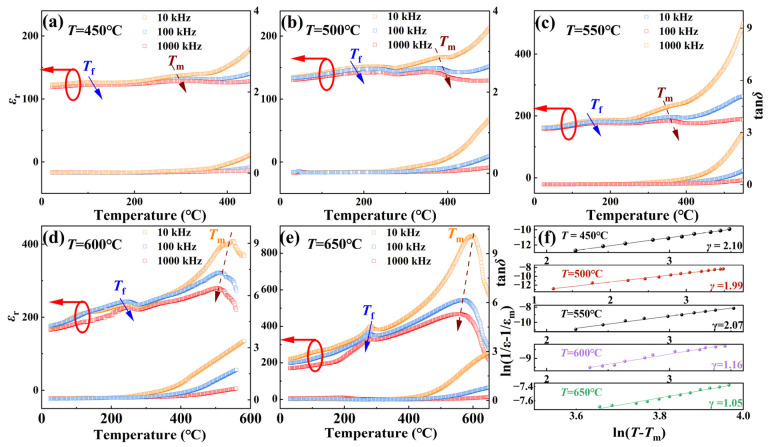
Temperature-dependent dielectric characteristics of NBTAO films acquired at varying crystallization temperatures, along with the corresponding fitted *γ* parameters.

Figure 3a–e presents the temperature-dependent dielectric constant (*ε*_r_) and loss tangent (tan*δ*) of NBTAO-450, NBTAO-500, NBTAO-550, NBTAO-600, and NBTAO-650 films measured across a range of frequencies. The *T*_f_ peak corresponds to the relaxation freezing temperature, a key signature of the transition from weak to strong RFE behavior [26]. As the crystallization temperature decreases, the *T*_f_ peak shifts to lower temperatures, indicating a strengthening of the material’s RFE behavior. The *T*_m_ peak corresponds to the ferroelectric-to-paraelectric phase transition temperature [27,28,29]. As *T*_m_ shifts progressively to lower temperatures, the NBTAO film’s RFE behavior strengthens. Concurrently, Raman spectroscopy indicates that a decrease in crystallization temperature enhances structural symmetry, specifically, tetragonality.

Frequency dispersion intensifies with decreasing crystallization temperature, reflecting enhanced RFE behavior. In the NBTAO-600 film, frequency dispersion is observed at *T*_f_ but absent at *T*_m_; in the NBTAO-650 film, it is absent at both peaks. This likely reflects a structural evolution from the ferroelectric to the RFE phase upon decreasing crystallization temperature. To corroborate this interpretation, the relaxation coefficient *γ* was fitted by the Curie–Weiss law [3,4,26].

(1) $$\frac{1}{\varepsilon_{r}}-\frac{1}{\varepsilon_{m}}=\frac{1}{C}(T-T_{m}{)}^{\gamma }$$

In the equation, C denotes the Curie constant and *γ* represents the relaxation diffusion coefficient. The Figure 3f show the relaxation diffusion coefficients *γ* for the NBTAO-450, NBTAO-500, NBTAO-550, NBTAO-600, and NBTAO-650 films, extracted from dielectric measurements at 10 kHz. The *γ* increases from ~1 to ~2 upon decreasing the crystallization temperature from 650 to 450 °C, signifying a structural evolution from the ferroelectric to the RFE phase. Low crystallization temperature suppresses long-range ferroelectric order and favors the emergence of dynamically disordered polar nanoregions (PNRs) [30]. Moreover, decreasing crystallization temperature reduces grain size and increases grain boundary density, both of which impede domain-wall motion and promote nucleation of antiparallel polarized domains, thereby enhancing the formation of disordered PNRs [31]. Crystallization temperature is a key process parameter for tailoring ferroelectric domain structure and thereby tuning RFE properties.

**Figure 4:**
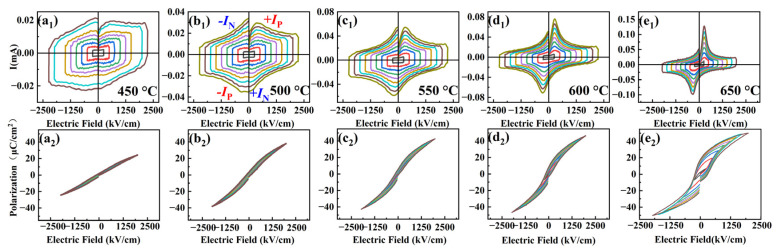
(**a_1_**–**e_1_**) The *P*–*C* curves of NBTAO-450, NBTAO-500, NBTAO-550, NBTAO-600, and NBTAO-650 films; (**a_2_**–**e_2_**) the *P*–*E* hysteresis loops at the same electric field.

Figure 4 shows the *P*–*C* curves and *P*–*E* hysteresis loops of NBTAO-450, NBTAO-500, NBTAO-550, NBTAO-600, and NBTAO-650 films, revealing how crystallization temperature affects RFE behavior. The two reverse current peaks in Figure 4a_1_–e_1_, along with the temperature-dependent dielectric response, demonstrate that lowering the crystallization temperature drives a transformation from the ferroelectric (FE) state to a non-ergodic relaxor (NER) state. Moreover, Figure 4b_1_ reveals that a further reduction in crystallization temperature induces a distinct four-peak current response in the NBTAO film, signifying a transition of its relaxation behavior from the NER to the ergodic relaxor state (ER) [26]. The forward peak (+*I*_P_) corresponds to the electric-field-induced transition from the ER to FE state, driven by the alignment and growth of ER nanodomains into long-range-ordered FE domains, accompanied by a sharp rise in orientation polarization. The reverse peak (+*I*_N_) reflects domain collapse upon field removal, reverting to short-range random nanodomains and markedly reducing hysteresis [26]. As the crystallization temperature is further reduced, the distinct four-peak current response observed in Figure 4b_1_ progressively broadens and attenuates (Figure 4a_1_), signifying the emergence of phase coexistence between amorphous and crystalline domains, resulting in substantial suppression of both current peak amplitude and polarization intensity. A decrease in crystallization temperature drives a hierarchical structural phase transition in the film: sequential evolution from the FE state, through the NER state and the ER state, to a coexisting crystalline and amorphous phase state. This phenomenon is intimately linked to both the crystal structure phase transition from the orthorhombic to the tetragonal phase and the concomitant grain size reduction, factors that jointly govern domain structure evolution and exert dominant control over the observed behavior. As shown in Figure 4a_2_–e_2_, the *P*–*E* hysteresis loops measured under identical electric-field amplitudes further corroborate the aforementioned structural phase transition. The *P*_max_ gradually diminishes with decreasing crystallization temperature and exhibits a pronounced reduction in the NBTAO-450 film. With decreasing crystallization temperature, the *P*–*E* hysteresis loops progressively narrow, and the *P*_r_ exhibits a monotonic decline. The coupled evolution of crystal structure and ferroelectric properties synergistically enhances the material’s energy storage performance.

**Figure 5:**
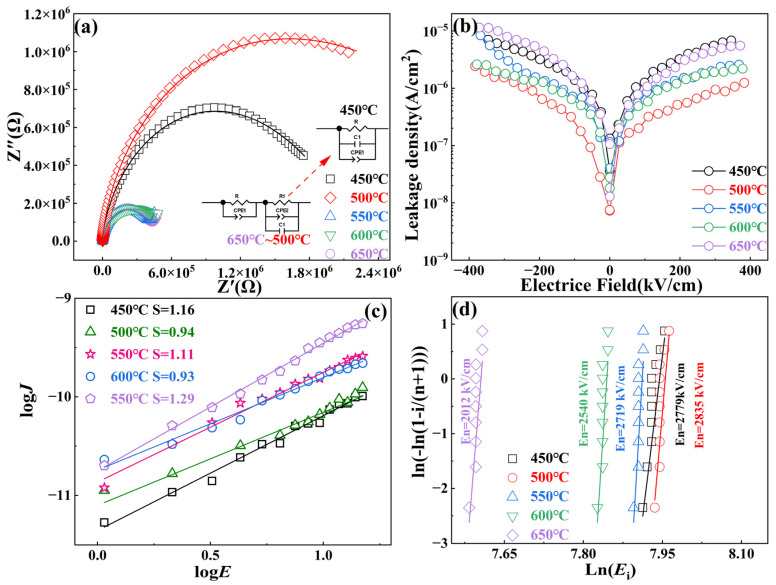
(**a**) Complex impedance results of NBTAO-450, NBTAO-500, NBTAO-550, NBTAO-600, and NBTAO-650 films; (**b**) leakage current density of the NBTAO films; (**c**) leakage current mechanism of the NBTAO films; and (**d**) Weibull distribution of NBTAO films.

Breakdown strength, like RFE characteristics, critically affects energy storage performance. Figure 5a shows the complex impedance spectra of the NBTAO-450, NBTAO-500, NBTAO-550, NBTAO-600, and NBTAO-650 films, measured from 100 Hz to 1 MHz at 450 °C. The semicircles observed in the complex impedance spectra, appearing in the high-frequency, mid-frequency, and low-frequency regions, are conventionally ascribed to the electrode/film interface, grain boundaries, and grain interior, respectively [32]. The complex impedance of the NBTAO-500, NBTAO-550, NBTAO-600, and NBTAO-650 films exhibit a depressed semicircular feature, reflecting overlapping contributions from grain interior and grain boundary conduction processes. In contrast, the complex impedance spectra of NBTAO-450 film exhibit well-defined, near-ideal semicircular arcs, attributable solely to grain interior conduction, with a negligible contribution from grain boundaries.

Notably, under identical test conditions, a systematic decrease in crystallization temperature leads to a progressive increase in the diameter of the semicircular arc in the complex impedance spectrum, directly reflecting a pronounced enhancement in bulk resistance. However, the NBTAO-450 film exhibits an anomalous reduction in impedance arc diameter, a deviation attributed to excessive suppression of crystallinity during annealing, resulting in a heterogeneous microstructure comprising both crystalline domains and amorphous regions, which collectively promote defect formation. As shown in Figure 5a, the complex impedance spectra of NBTAO-450, NBTAO-500, NBTAO-550, NBTAO-600, and NBTAO-650 films, acquired at identical frequency ranges and under controlled DC bias conditions, were modeled using equivalent circuits implemented in Zview software(V3.3). The impedance response of the NBTAO film exhibits excellent agreement with the experimental spectra. Accordingly, the impedance data were modeled using an equivalent circuit comprising a series connection of R//C//CPE subcircuits and R//CPE at crystallization temperatures above 500 °C (inset of Figure 5a). Upon lowering the crystallization temperature, the dominant relaxation process simplifies to a single (R//C//CPE) subcircuit, where R denotes resistance, C denotes capacitance, and CPE denotes the constant phase element. High-density grain boundaries with elevated resistivity significantly enhance the electrical insulation performance of NBTAO film. An optimized crystallization temperature markedly improves the dielectric breakdown strength of films. However, an excessively low crystallization temperature will lead to an increase in defect density, thereby weakening the dielectric breakdown strength. The leakage current density–electric field characteristics in Figure 5b quantitatively demonstrate the improved insulation performance. At room temperature and an applied electric field of 370 kV/cm, NBTAO-500 film exhibits a leakage current density of 1.24 × 10^−6^ A/cm^2^, lower than that of NBTAO-650 (5.53 × 10^−6^ A/cm^2^). Upon further reduction of the crystallization temperature, the leakage current density of NBTAO-450 film exhibits an anomalous increase. Figure 5c summarizes the leakage conduction mechanism analysis, demonstrating that Ohmic conduction governs the leakage current response across all NBTAO films, which is characterized by ln(J) ∝ ln(E) with a slope of approximately 1, irrespective of crystallization temperature. Figure 5d summarizes the Weibull distribution-derived breakdown field strength (*E*_b_) of all NBTAO films, with the NBTAO-500 sample exhibiting an *E*_b_ of 2835 kV/cm [33,34,35,36,37]. Lowering the crystallization temperature induces grain refinement, which increases the volumetric proportion of high-resistance grains and impedes long-range charge carrier transport, thereby elevating the intrinsic breakdown strength. Concurrently, high-density grain boundaries featuring elevated electrical resistance act as potent scattering centers for charge carriers (e.g., electrons and oxygen vacancies), retarding percolation-driven breakdown path formation. Collectively, these microstructurally engineered effects synergistically enhance the effective *E*_b_.

**Figure 6:**
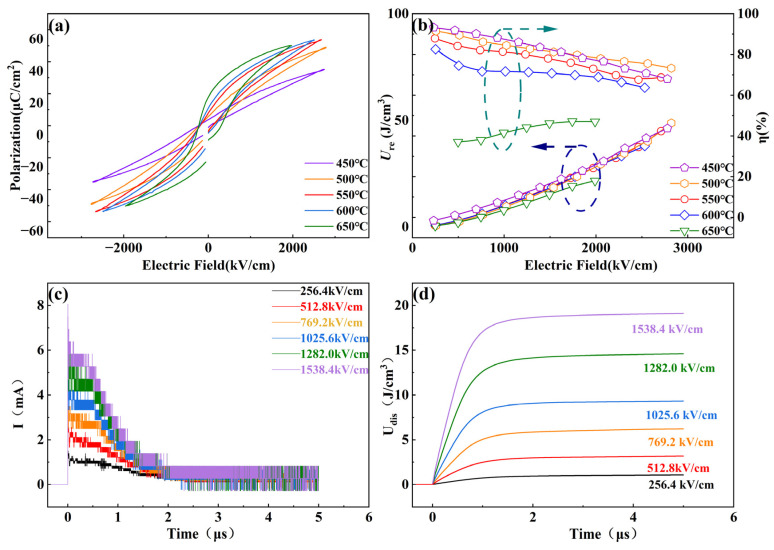
(**a**) *P*–*E* hysteresis loops measured at the maximum electric field. (**b**) The *U*_re_ and *η* of NBTAO-450, NBTAO-500, NBTAO-550, NBTAO-600, and NBTAO-650 films. (**c**) The discharge current curve of NBTAO-500 films at RT. (**d**) The *U*_dis_ of NBTAO-500 films under different electric fields.

Figure 6a presents the *P*–*E* hysteresis loops of NBTAO-450, NBTAO-500, NBTAO-550, NBTAO-600, and NBTAO-650 films, measured at *E*_b_. As the crystallization temperature decreases, the *P*–*E* hysteresis loops of the NBTAO films progressively narrow, reflecting enhanced RFE behavior induced by the lower crystallization temperature. The *P*_max_ of NBTAO-450, NBTAO-500, NBTAO-550, NBTAO-600, and NBTAO-650 films are 35.1, 48.8, 53.8, 53.7 and 50.2 μC/cm^2^, respectively. The reduction in crystallization temperature enhances the *E*_b_ of the NBTAO films, thereby delaying the onset of saturation polarization and enabling effective retention of the *P*_max_. The *U*_re_ and *η*, calculated from the data in Figure 6a, are presented in Figure 6b. The NBTAO-500 film achieves the highest *U*_re_ and *η* at the maximum applied electric field, owing to the synergistic enhancement of dielectric relaxation behavior, breakdown strength, and resistance to polarization saturation. *U*_re_ and *η* reach 49.6 J/cm^3^ and 73.5%, respectively, at an applied electric field of 2820 kV/cm. While the NBTAO-450 film also exhibits favorable energy storage performance, its deteriorated insulation capability leads to a concurrent decline in both *U*_re_ and *η*. The energy density and efficiency of the NBTAO-500 film not only outperform those of NBTAO films annealed at other temperatures but also exceed those of previously reported representative lead-free material systems and Aurivillius-type films, as depicted in Figure S2 [4,13,14,21,38,39,40,41,42,43,44,45]. The discharge energy density (*U*_dis_) of the NBTAO-500 film was quantified via pulsed charge–discharge measurements. As shown in Figure 6c, the discharge current response of the NBTAO-500 film at room temperature exhibits a rapid rise under applied electric fields ranging from 256.4 to 1538.4 kV/cm, reaching its peak within <1.5 μs. *U*_dis_ was calculated using the following equation:

(2) $$U_{\mathrm{dis}}=\frac{R\int {I(t)}^{2}\mathrm{dt}}{V}$$

The load resistance is designated as *R* (2000 Ω), the volume as *V*, and the *U*_dis_ is presented in Figure 6d. As the applied electric field increases from 256.4 to 1538.4 kV/cm, the peak discharge current (*I*_max_) rises linearly, whereas *U*_dis_ follows a parabolic trend, increasing from 1.02 to 19.0 J/cm^3^. Furthermore, the NBTAO-500 film enables ultrafast discharge, with full discharge completed within <1.5 μs across the entire field range. The deviation between *U*_dis_ and *U*_re_ remains within ~10%, confirming the high fidelity of the indirect energy estimation method, in agreement with prior reports [25].

In practical applications, particularly those demanding high operational stability, dielectric energy storage capacitors require robust temperature and frequency stability.

**Figure 7:**
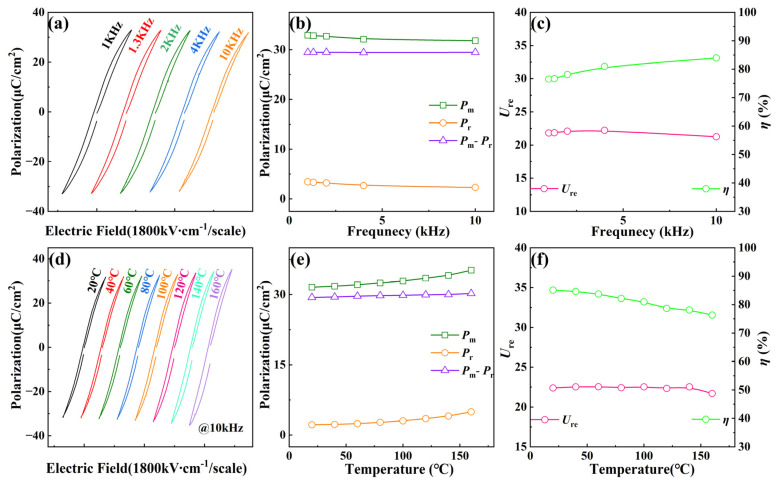
(**a**) *P*–*E* hysteresis loops of NBTAO-500 film at different frequencies; (**b**) *P*_m_, *P*_r_ and *P*_m_–*P*_r_ of NBTAO-500 film at different frequencies; (**c**) *U*_re_ and *η* of NBTAO-500 film at different frequencies; (**d**) *P*–*E* hysteresis loops of NBTAO-500 film at different temperatures; (**e**) *P*_m_, *P*_r_ and *P*_m_–*P*_r_ of NBTAO-500 film at different temperatures; and (**f**) *U*_re_ and *η* of NBTAO-500 film at different temperatures.

Figure 7a displays the *P*–*E* hysteresis loops of the NBTAO-500 film measured at 1754 kV/cm across a frequency range of 1~10 kHz. With increasing frequency, the loops exhibit progressively narrower shapes, reflecting reduced dielectric loss and enhanced high-frequency charge-discharge efficiency, consistent with the intrinsic dynamic response of relaxor ferroelectrics. As illustrated in Figure 7b, parameters *P*_m_, *P*_r_, and *P*_m_–*P*_r_ exhibit negligible variation across the tested frequency range, confirming their outstanding frequency stability. Figure 7c presents the frequency-dependent energy storage performance of the NBTAO-500 film. Over the 1~10 kHz range, *U*_re_ remains nearly constant at approximately 21.8–21.3 J/cm^3^, while the *η* increases from 76.5% to 83.9%. These minimal variations confirm the film’s exceptional frequency stability. Figure 7d presents the *P*–*E* hysteresis loops of the NBTAO-500 film, measured from 20~160 °C under a constant applied electric field of 1754 kV/cm. As shown in Figure 7e, as temperature increases, the *P*–*E* hysteresis loops retain their narrow, slender morphology; concurrently, both the maximum polarization *P*_m_ and *P*_r_ exhibit slight increases, whereas the difference (*P*_m_–*P*_r_) remains virtually constant across the tested temperature range, demonstrating the exceptional thermal stability of the film’s polarization response. Figure 7f presents the temperature-dependent energy storage performance of the NBTAO-500 film. Over the 20~160 °C range, the *U*_re_ decreases modestly from 22.39 to 21.69 J/cm^3^, while the *η* declines from 85.1% to 77.5%. These minimal variations confirm the film’s exceptional thermal stability. The NBTAO-500 film demonstrates exceptional thermal stability across the tested temperature range. The results demonstrate that the NBTAO-500 film exhibits exceptional thermal and frequency stability. Across a broad range of temperatures and frequencies, it consistently maintains high *U*_re_ and *η*, underscoring its strong promise for energy storage applications.

## 4. Conclusions

In summary, crystallization temperature was systematically tuned to tailor both the crystal structure and relaxor behavior of NBTAO films. A crystallization temperature-dependent structure property regulation pathway has been established, enabling systematic and controllable transitions from the ferroelectric state, through the non-ergodic relaxor state, to the ergodic relaxor state. Precise control of the crystallization temperature enables grain size reduction, which concurrently enhances the bulk resistivity and breakdown strength, two key dielectric properties intrinsically linked through suppressed conduction pathways. The NBTAO-500 film ultimately achieves excellent energy storage performance, delivering a *U*_re_ of 49.6 J/cm^3^ and an *η* of 73.5% at an applied electric field of 2820 kV/cm. The NBTAO-500 film also demonstrates excellent thermal and frequency stability. This establishes crystallization temperature control as a viable strategy for enhancing energy storage performance, thereby expanding the multidimensional optimization pathways for dielectric energy storage films.

## Figures and Tables

**Table 1:** Source materials information.

Original Material	Material Purity	Manufacturer
Bi(NO_3_)_3_·5H_2_O	99%	Innochem; Beijing; China
Al(NO_3_)_3_·9(H_2_O)	99%	Innochem; Beijing; China
C_2_H_3_NaO_2_	99.9%	Innochem; Beijing; China
C_16_H_36_O_4_Ti	98+%	Innochem; Beijing; China
C_3_H_8_O_2_	99.5%	Innochem; Beijing; China
C_5_H_8_O_2_	99.0%	Innochem; Beijing; China
Pt(111)/Ti/SiO_2_/Si(100)	--------	Institute of Microelectronics, Peking University; Beijing; China

**Table 2:** Instrument information.

Instrument Name	Producing Country	Manufacturer
Electronic balance (SQP)	Göttingen; Germany	Sartorius
Spin coater (EZ4-S)	Wuxi; China	Lebo science
Rapid annealing system (AS-Micro RTP System)	Montpellier; France	Annealsys
X-Ray Diffraction (D/MAX-2500/PC)	Tokyo; Japan	Rigaku
Raman spectrometer (DXR2Xi)	Waltham, MA; USA	ThermoFisher Scientific
Scanning electron microscopy (SU8220)	Tokyo; Japan	Hitachi
Magnetron sputtering instrument (ISC150)	Shenzhen; China	SuPro Instruments
Multiferroic test system (MultiFerroic 500 V)	Albuquerque, NM; USA	Radiant
LCR digital bridge (E4980AL)	Santa Rosa, CA; USA	Keysight
High-temperature probing stage (GWDS003)	Shanghai; China	Tongguo Technology
High-temperature probing stage (HCT1821)	Shanghai; China	Tongguo Technology

## Data Availability

The raw data supporting the conclusions of this article will be made available by the authors on request.

## Supplementary Materials

The following supporting information can be downloaded at https://www.mdpi.com/article/10.3390/nano16130802/s1, Figure S1: SEM surface images and particle size distributions of the Na_0.5_Bi_5.5_Ti_4_AlO_18_ film at different annealing temperatures; Figure S2: Comparison of *U*_re_/E among different films.
