# Supplementary material for: Tailoring High Energy Storage Density by a Temperature-Induced Relaxor-to-Ferroelectric Phase Transition
Source: Nanomaterials (Basel). 2026 Jun 29;16(13):802. doi: 10.3390/nano16130802 (PMC13363211; doi:10.3390/nano16130802)
Supplement: Supplementary file 1 [file nanomaterials-16-00802-s001.zip › nanomaterials-4386834-supplementary.pdf]

Supporting information

Tailoring High Energy Storage Density by a Temperature-Induced Relaxor-to-Ferroelectric Phase Transition

Qiang Lv [1,2] and Jieyu Chen [1,2,3,*]

[1] College of Science, Inner Mongolia University of Technology, Hohhot 010051, China; 202320908044@imut.edu.cn
[2] Discharge Plasma and Functional Materials Application Laboratory, Inner Mongolia University of Technology, Hohhot 010051, China
[3] Inner Mongolia Key Laboratory of New Materials and Surface Engineering, School of Materials Science and Engineering, Inner Mongolia University of Technology, Hohhot 010051, China
* Correspondence: 20190000100@imut.edu.cn

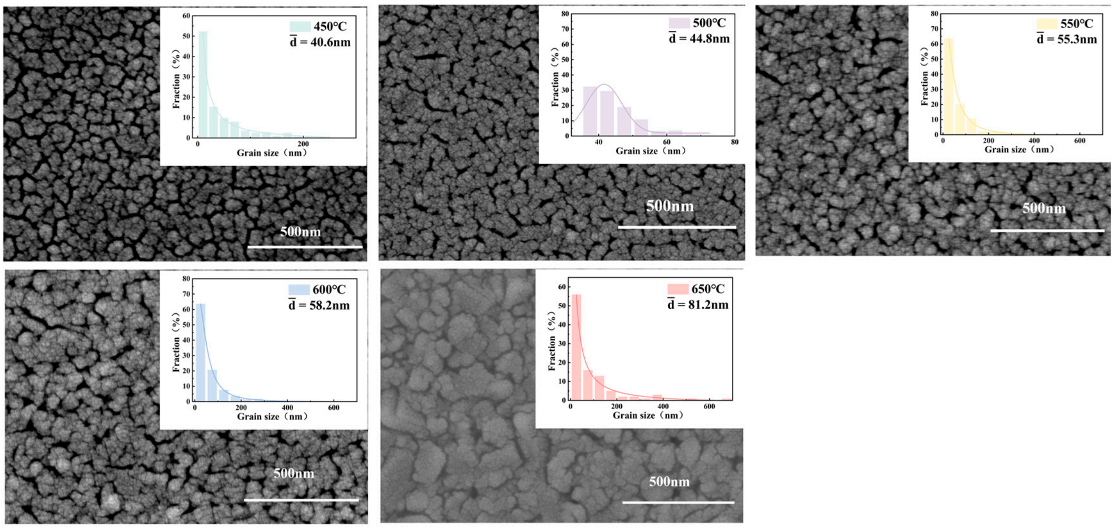

Figure S1. SEM surface images and particle size distributions of the $Na_{0.5}Bi_{5.5}Ti_4AlO_{18}$ film at different annealing temperatures.

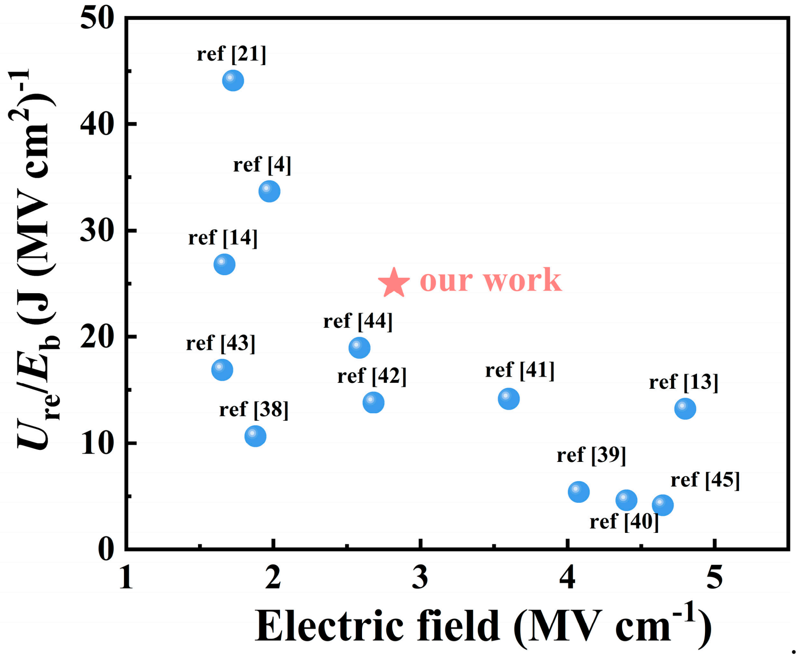

Figure S2. Comparison of U_{re}/E among different films.
